# Fabrication of High‐Density Microarchitected Tungsten via DLP 3D Printing

**DOI:** 10.1002/advs.202405487

**Published:** 2024-08-13

**Authors:** Junyu Cai, Songhua Ma, Wenbin Yi, Jieping Wang

**Affiliations:** ^1^ School of Chemistry and Chemical Engineering Nanjing University of Science and Technology Nanjing 210094 China

**Keywords:** additive manufacturing, digital light processing, high‐density, high‐hardness, tungsten

## Abstract

Current additive manufacturing (AM) techniques for tungsten, such as powder bed fusion and directed energy deposition, often generate parts with rough surfaces. Vat photopolymerization presents a promising alternative for fabricating tungsten structures with high shape fidelity and low surface roughness. However, existing vat photopolymerization approaches suffer from surface defects and low final density, leading to compromised mechanical properties. Therefore, achieving high‐density tungsten structures using vat photopolymerization remains a crucial challenge. This work presents a straightforward and reliable method for fabricating complex, micro‐architected tungsten structures with superior density and hardness. The approach utilizes a water‐based photoresin with exceptional tungsten ion loading capacity. The photoresin is then patterned using digital light processing (DLP) to create tungsten‐laden precursors. A three‐step debinding and sintering process subsequently achieves 3D tungsten structures with dense surface morphology and minimal internal defects. The microstructures achieve a minimum feature size of 35 µm, a low surface roughness of 2.86 µm, and demonstrate exceptional mechanical properties. This new method for structuring tungsten opens doors to a broad range of applications, including micromachining, collimators, detectors, and metamaterials.

## Graphical abstract

This study introduces a straightforward and reliable method for producing high‐density micro‐architected tungsten structures, characterized by a minimum feature size of 35 µm, low surface roughness of 2.86 µm, and outstanding mechanical properties. This innovative approach paves the way for a diverse array of applications, encompassing micromachining, collimators, detectors, and metamaterials.

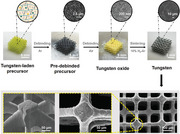

## 1 Introduction

Tungsten (W) is highly valued for its distinctive combination of properties, including high density, exceptional thermal and electrical conductivity, remarkable mechanical strength, and superior chemical stability.^[^ ^1^ ^]^ These attributes make tungsten a valuable material across various industries, where it finds applications in microelectrode,^[^ ^2^ ^]^ MEMS devices,^[^ ^3^ ^]^ plasma‐facing components,^[^ ^4^ ^]^ collimators and detectors for medical computed tomography (CT) machines,^[^ ^5^ ^]^ and radiation shielding components.^[^ ^6^ ^]^ However, unlocking the full potential of tungsten hinges on its internal structure. Pores created during processing can significantly reduce its effectiveness. For instance, lower density compromises properties like radiation shielding ability, thermal conductivity, and compressive strength. While full‐densification tungsten can be achieved through conventional methods such as powder metallurgy,^[^ ^7^ ^]^ chemical vapor deposition,^[^ ^8^ ^]^ and vacuum melting,^[^ ^9^ ^]^ these processes typically entail demanding setups, complex procedures, and stringent conditions. Moreover, they often have limitations in producing complex geometries, thereby constraining their utility in applications requiring complex structures.

Additive manufacturing (AM) offers a promising path toward creating tungsten structures with complex geometries.^[^ ^10^ ^]^ However, existing AM techniques for tungsten, such as powder bed fusion^[^ ^11^ ^]^ and directed energy deposition,^[^ ^12^ ^]^ face limitations due to beam size and powder quality. These limitations can result in rough or uneven surfaces on fabricated tungsten parts.^[^ ^13^ ^]^ While direct ink writing of a tungsten (VI) oxide‐containing ink presents an alternative approach for tungsten fabrication, achieving high‐resolution features with this method remains challenging.^[^ ^14^ ^]^ Consequently, these limitations impede the ability of the aforementioned AM techniques to extend their application scope to the domain of microdevice manufacturing.

Vat photopolymerization offers a promising avenue for fabricating complex metallic structures with superior shape fidelity and lower surface roughness compared to other additive manufacturing techniques.^[^ ^15^ ^]^ Subsequent heat treatment can further refine the microstructure, making it particularly advantageous for microdevice manufacturing. Two‐photon lithography (TPL) and digital light processing (DLP) have been employed for tungsten fabrication. However, current methodologies, such as DLP explored by Greer^[^ ^15a^ ^]^ and Wang,^[^ ^15d^ ^]^ and TPL demonstrated by Luitz,^[^ ^15e^ ^]^ encounter significant limitations. These include surface defects and low density in the fabricated tungsten, resulting in compromised mechanical strength. Notably, the maximum reported stress tolerance for these light‐cured, porous tungsten structures is a mere 0.9 MPa,^[^ ^15e^ ^]^ insufficient for load‐bearing applications. Therefore, developing a method to achieve high‐density tungsten structures using vat photopolymerization remains a critical goal.

**Figure 1:**
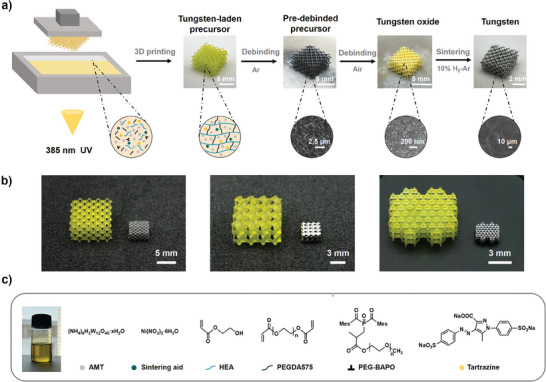
a) Fabrication process for high‐density tungsten microstructures. b) Printed precursors (left) and sintered tungsten (right). c) Tungsten‐containing photoresin formulation.

Herein, we report an innovative vat photopolymerization method for fabricating complex, micro‐architected tungsten structures with superior density and hardness (**Figure** **1**a). This approach utilizes a water‐based photoresin containing a high concentration of tungsten ions (47.8 wt.%). The photoresin is patterned using DLP to generate complex tungsten‐laden precursors. A three‐step debinding and sintering process enables the successful creation of 3D tungsten structures with a dense surface morphology and minimal internal defects (Figure 1b). These microstructures exhibit a minimum feature size of ≈35 µm, a low surface roughness of 2.86 µm, and exhibit exceptional mechanical properties.

## 2 Results and Discussion

A water‐based photoresin was formulated, comprising ammonium metatungstate (AMT) as the tungsten source, nickel nitrate hexahydrate (Ni(NO_3_)_2_·6H_2_O) as a sintering aid, acrylic acid‐2‐hydroxyethyl (HEA) as an active diluent, polyethylene glycol diacrylate (PEGDA 575) as a cross‐linker, PEG‐BAPO**^[^** ^16^ **^]^** as a highly efficient waterborne photoinitiator, and tartrazine as a light‐absorber (Figure 1c). Initially, precursors with varying AMT content were prepared by patterning a range of photoresins with different AMT concentrations (Table S1, Supporting Information). Increasing the loading of AMT resulted in reduced surface defects and enhanced structural integrity in both debinded and sintered samples (Figure S1, Supporting Information). To maximize the AMT content while maintaining appropriate viscosity for patterning, photoresins with different PEGDA 575 to HEA volume ratios were then investigated (Table S1, Supporting Information). Through precise adjustment of the PEGDA 575 to HEA ratio (Figure S2, Supporting Information), a photoresin with low viscosity (0.4645 Pa·s) was achieved. The tungsten content of the photoresin reached 47.8 wt.%, surpassing that of relevant studies by a factor of two (Figure S3, Supporting Information). Despite significant absorption by AMT at 385 nm (Figure S4, Supporting Information), the photoinitiator demonstrated effective performance in photopolymerization (Figure S5, Supporting Information).

The photoresin was subsequently patterned using digital light processing (DLP) at a wavelength of 385 nm and an exposure dose of 10 mW cm^−2^. Tartrazine significantly enhanced the printing resolution (Figure S6, Supporting Information). To maintain surface accuracy, a printing layer thickness of 10 µm was utilized, corresponding to an exposure time of 5.5 s (Table S2, Supporting Information). Scanning electron microscopy (SEM) revealed a dense morphology throughout both the surface and cross‐section of the cured precursor (Figure S7, Supporting Information).

**Figure 2:**
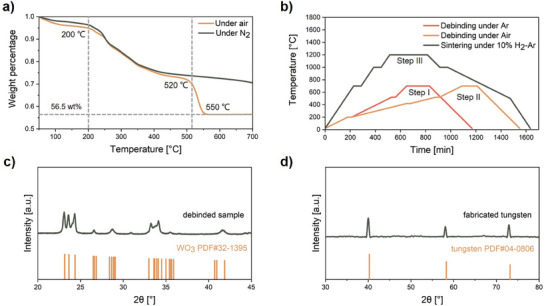
a) TGA of the tungsten‐laden precursor. b) Temperature ramping profile of the three‐step debinding and sintering process. c) XRD spectrum of the debinded sample. d) XRD spectrum of the sintered sample.

Thermogravimetric analysis (TGA) of the precursor (**Figure** **2**a) played a crucial role in optimizing the debinding and sintering process parameters. A relatively slow weight loss observed at the initial stage (20–200 °C) corresponds to the decomposition of free water (inherent to the photoresin) and crystalline water (associated with AMT). From 200 to 500 °C, a significant increase in the weight‐loss rate is observed, corresponding to the combined decomposition of AMT and organic polymers within the precursor. AMT decomposes completely into WO_3_ at 500 °C.**^[^** ^17^ **^]^** Beyond 500 °C, the decomposition of organic polymers exhibits varying weight‐loss rates depending on the surrounding atmosphere.

**Figure 3:**
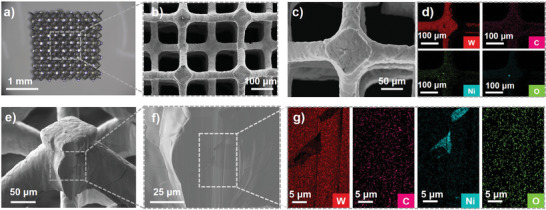
a) An optical image of the fabricated tungsten microstructure. b) SEM images of the fabricated tungsten microstructure. c) The surface characteristics of the fabricated tungsten. d) EDX mapping of Figure 3c. e,f) Cross‐section of the fabricated tungsten. g) EDX mapping of Figure 3f.

A three‐step process facilitated the transformation of the precursor into dense tungsten (Figure 2b). The debinding process involved the removal of organic polymers and the decomposition of AMT into WO_3_. Initially, inspired by Greer,**^[^** ^15a^ **^]^** printed precursors were directly debinded in air. However, it was observed that structures with finer features experienced partial structural collapse during direct air debinding. This phenomenon can be attributed to the intense decomposition of polymers occurring at temperatures between 520 and 550 °C when heating in air. In contrast, the weight loss of the precursor in an inert atmosphere exhibited a considerably more gradual pattern (Figure 2a). The slower rate of thermal decomposition in the inert atmosphere slowed the escape of gases, thereby reducing the probability of structural collapse. Therefore, the debinding strategy was modified by implementing an initial debinding step under an argon atmosphere followed by debinding in air. This sequential approach minimized cracking compared to direct air debinding (Figure S8, Supporting Information). During the primary weight‐loss stage, slower heating rates (1 °C min^−1^ under argon and 0.5 °C min^−1^ under air) were employed to control the release of decomposition gases and minimize pore formation. Isothermal holds of 0.5 h each were implemented at 220, 420, and 520 °C, corresponding to TGA inflection points. Energy‐dispersive X‐ray spectroscopy (EDX) revealed the presence of 22.4 wt.% carbon in the pre‐debinded precursor, indicating the residual presence of polymers (Figure S9, Supporting Information). Furthermore, X‐ray diffraction (XRD) analyzes (**Figure** **3**c) and Energy‐dispersive X‐ray spectroscopy (EDX) (Figure S10d, Supporting Information) confirmed the formation of WO_3_ following debinding. SEM analysis revealed a relatively dense WO_3_ surface with a particle size of ≈50 nm (Figure S10c, Supporting Information).

After debinding, the WO_3_ underwent reduction and sintering under a 10% H_2_‐Ar atmosphere. Reduction occurred at 700 °C for 1 h, followed by sintering at a relatively low temperature of 1200 °C for 5 h (Figure 2b). Notably, Ni(NO_3_)_2_·6H_2_O underwent a transformation into nickel, which acted as an effective sintering activator during thermal decomposition and reduction, significantly enhancing tungsten densification during sintering.**^[^** ^18^ **^]^**

**Figure 4:**
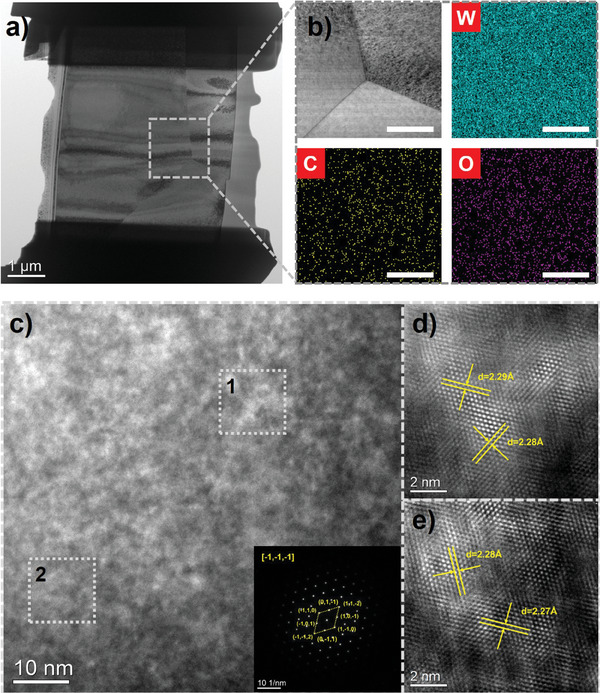
a) TEM images of FIB‐ed sample. b) STEM‐EDX of Figure 4a. c) HRTEM image and SAED pattern of the fabricated tungsten. d,e) HRTEM image obtained from Figure 4c.

Dense tungsten microstructures with complex geometries were fabricated, achieving a minimum feature size of 35 µm (Figure 3). Notably, the fabricated structures exhibited isotropic shrinkage, with a linear shrinkage factor of ≈40% across all axes (Figure S11, Supporting Information), closely matching the predicted theoretical value of 38.5% (Discussion S1, Supporting Information). This minimal and isotropic shrinkage signifies high‐density tungsten microstructures. Brunauer–Emmett–Teller (BET) surface area analysis revealed limited adsorption characteristics, suggesting a low specific surface area and minimal microporosity (Figure S12, Supporting Information). SEM images confirmed exceptional density with minimal pores or cracks on both the surface and cross‐section (Figure 3c–f). This result is significant as most vat photopolymerization techniques for tungsten yield porous structures.^[^ ^15d^ ^,^ ^15e^ ^]^ X‐ray diffraction (XRD) (Figure 2d) and Energy‐dispersive X‐ray spectroscopy (EDX) (Figure S13, Supporting Information) analysis revealed high‐purity tungsten with minor surface contamination. The surface composition was 95.31 wt.% W, 0.48 wt.% Ni, 2.30 wt.% C, and 1.91 wt.% O, while the cross‐section had a higher W content (96.76 wt.%) and lower levels of C and O. Nickel‐rich areas were observed in the cross‐section, consistent with previous report.^[^ ^14^ ^]^ Remarkably, a significant quantity of dislocations was observed in the FIB‐ed sample (**Figure** **4**a), a phenomenon typically associated with plastic deformation or cold working. Transmission electron microscopy (TEM) analysis revealed strong cohesion between tungsten grains with no boundary pores (Figure 3a). W, C, and O elements were uniformly distributed within grains and at grain boundaries (Figure 3b). The electron diffraction pattern and subsequent Fast Fourier Transform (FFT) analysis confirmed the single‐crystalline nature of the 3D‐printed tungsten, with interatomic spacings consistent with standard tungsten references (Figure 3c–e). No evidence of amorphous carbon or WO_3_ was detected, suggesting dissolved C and O within the tungsten lattice. Finally, electron backscatter diffraction (EBSD) revealed a random grain orientation with an average size of 18.8 µm, ranging from 5.6 to 42.5 µm (Figure S14, Table S3, Supporting Information).

**Figure 5:**
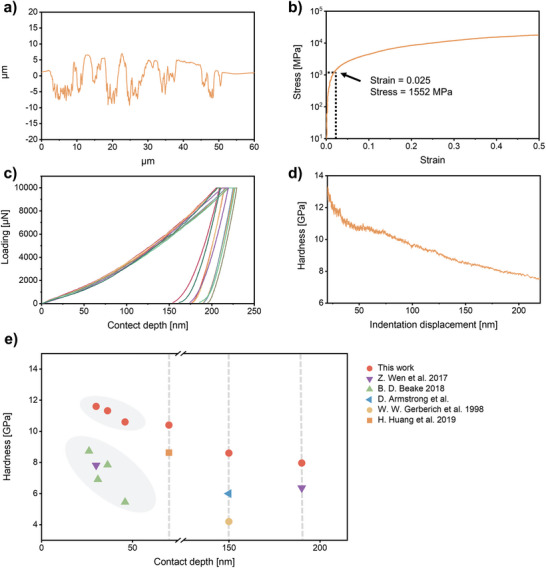
Characterization of the fabricated tungsten microstructure: a) Surface roughness analysis, b) Stress–strain curve of a 6 µm diameter micropillar, c) Load‐displacement curve measured in load‐controlled mode, d) Dependence of nanohardness on indentation depth, and e) Comparison of nanoindentation hardness with previously reported works.

The fabricated tungsten exhibited a surface roughness (*R*_a_) of 2.86 µm (**Figure** **5**a), indicative of a smooth surface finish. Micropillar compression tests assessed the local mechanical properties of the fabricated tungsten (Figure S15, Supporting Information). The stress–strain curve indicated a yield strength of 1552 MPa (Figure 5b), which agreed well with the predicted value (1585 MPa, Discussion S2, Supporting Information). This suggested a similar local compressive strength to bulk tungsten. Nanoindentation testing further investigated mechanical properties. The load‐controlled mode exhibited good repeatability (Figure 5c). At 10 mN load, the fabricated tungsten displayed an average hardness of 7.5 GPa and Young's modulus of 292.2 GPa (Table S5, Supporting Information). To investigate hardness variation, continuous stiffness mode (CSM) was employed (Figure 5d). Despite a hardness gradient (13.2–7.6 GPa) with increasing depth (20–220 nm), the fabricated tungsten exhibited superior hardness throughout the tested range compared to commercially available tungsten. Notably, it surpassed reference values by 1.36 to 2.05 times (Figure 5e; Table S6, Supporting Information). This could be attributed to the presence of dissolved light elements (such as C and O) within the lattice or at grain boundaries,^[^ ^15^, ^19^ ^]^ along with the roughness of grain boundaries caused by grain growth and interlocking upon reduction.^[^ ^20^ ^]^ Furthermore, the substantial presence of dislocations in the fabricated tungsten also contributed to the enhancement of its properties.^[^ ^21^ ^]^ To evaluate the bulk mechanical properties, a compression test revealed a remarkable compressive strength of 13 MPa for the fabricated tungsten lattice (Figure S16, Supporting Information). This value represents a significant enhancement compared to the previously reported value of only 0.9 MPa, demonstrating a substantial improvement in mechanical performance.^[^ ^15e^ ^]^

In summary, this work presents a novel approach for fabricating complex tungsten microstructures with superior mechanical properties using vat photopolymerization. We unveil the previously unexplored yet crucial role of high metal ion content in facilitating debinding and sintering. Leveraging this insight, we developed a water‐based photoresin with an exceptional tungsten ion concentration (47.8 wt.%). A pre‐debinding step is introduced to prevent collapse during debinding, offering a valuable strategy for future studies. The resulting microstructures boast a minimum feature size of 35 µm and a low surface roughness of 2.86 µm. Remarkably, these microstructures exhibit superior mechanical properties, including a micropillar compressive strength of 1552 MPa, nanoindentation hardness reaching 7.5 GPa, and an overall compressive strength of 13 MPa for an entire tungsten lattice. This innovative method has the potential to revolutionize various fields, including micromachining, collimation, detection, and metamaterial development.

## 3 Experimental Section

### Materials

Polyethylene glycol diacrylate (PEGDA, *M*_n_ = 575 g mol^−1^) was obtained from Sigma–Aldrich. Acrylic acid‐2‐hydroxyethyl (HEA) and tartrazine were purchased from Adamas. Ammonium metatungstate was acquired from Aladdin. Ni(NO_3_)_2_·6H_2_O was obtained from SCR. D‐RO water was purchased from Rhawn. PEG‐BAPO was synthesized according to established literature procedures.^[^ ^16^ ^]^

### Preparation of Tungsten‐Contained Organic–Inorganic Photoresin

A photoresin was prepared by combining the following components: 1) matrix (40 wt.%): composed of 20 vol% DI water, 30 vol% HEA, and 50 vol% PEGDA575; 2) ammonium metatungstate (60 wt.%); 3) PEG‐BAPO (0.5 wt.%); 4) tartrazine (0.05 wt.%). Ni(NO_3_)_2_·6H_2_O) was added at tungsten (W) to nickel (Ni) atomic ratio of 50:1. All components were mixed and stirred for 12 h to ensure complete dissolution.

### Printing of Tungsten‐Contained Precursors

The photoresin was patterned using DLP with a wavelength of 385 nm and an exposure dose of 10 mW cm^−^^2^ (Asiga Max X27 UV385). The exposure time was 5.5 s, corresponding to a printed layer thickness of 10 µm. After printing, the precursors were removed from the platform with a squeegee and rinsed with water and isopropyl alcohol to remove uncured photoresin. Further details on printing parameters can be found in Table S2 (Supporting Information).

### Thermal Treatment

The photoresin precursors underwent a three‐step thermal treatment process: 1) Pre‐debinding: conducted under argon (Ar) with a flow rate of 100 mL min^−1^ in a tube furnace (GSL‐1600X). 2) Debinding: carried out in air using a muffle furnace (KSL‐1100X‐S). 3) Sintering: performed under a 10% hydrogen (H₂) in argon (Ar) atmosphere with a flow rate of 100 mL min^−1^ in a tube furnace (GSL‐1600X). For detailed information on the thermal treatment parameters, refer to (Figure 2b).

### Material Characterization

Optical transparency and absorption of the tungsten‐contained photoresin were measured using a UV–vis spectrophotometer (Evolution220, Thermo Fisher). Rheological characteristics of the photoresins were conducted on a stress‐controlled rheometer (Kinexus Pro+) at 25 °C. Thermogravimetric Analysis (TGA) of the precursors was performed using a thermal gravimetric analyzer (NETZSCH STA 449F3) with a heating rate of 0.5 °C min^−1^ from room temperature to 700 °C. Tungsten‐laden precursors were sputter‐coated for further characterization. A V‐Sorb 2800TP surface area and porosity analyzer were employed to assess the porosity of tungsten samples using nitrogen gas adsorption. Scanning electron microscopy (SEM) images were obtained using two equipment: 1) a FIB/SEM dual‐beam system (Zeiss Auriga) with a 15 kV accelerating voltage; 2) scanning electron microscope (JSM‐7800F PRIME) with a 10 kV accelerating voltage. Cross‐sections of tungsten lattices were prepared using a gallium FIB within the same instrument at 30 kV and 50 nA. Energy‐dispersive X‐ray spectroscopy (EDX) analysis was performed using an Oxford 150 Max system with a 15 kV electron beam. X‐ray diffraction (XRD) was conducted using a Bruker D8 Advanced diffractometer with a Cu Kα source operating at 40 kV and 40 mA. Transmission electron microscopy (TEM) specimens were prepared using a Thermo Scientific Scios 2 SEM equipped with a Ga FIB (30 kV) and an Omniprobe manipulator. TEM imaging was performed on a TECNAL F30 microscope with a 300 kV beam. Electron backscatter diffraction (EBSD) was conducted using an Oxford EBSD system within a CIQTEK SEM5000 at 20 kV accelerating voltage with a 1.5 µm scanning step size. Kikuchi map data analysis was performed using AZtecHKL software. Surface roughness was measured using an optical profilometer (Bruker Contour GT‐K 3D).

### Mechanical Performance Testing

Tungsten micropillars with a diameter of 6 µm were prepared using a Ga focused‐ion beam (FIB) within a scanning electron microscope (SEM, Thermo Scientific Scios 2). The fabricated micropillars then underwent in situ compression testing using the same instrument. A diamond indenter with a 5 µm diameter was used for compression at a constant strain rate of 20 nm s^−1^ in displacement mode. Load‐displacement data was recorded simultaneously. Separately, tungsten samples were polished and tested using a nanoindentation instrument (Bruker Hysitron TI980) equipped with a Berkovich diamond tip with a half‐angle of 65.27°. The tests employed a load‐controlled mode with a maximum load of 10 mN and a continuous stiffness mode (CSM) with a 1 nm oscillation and a maximum indent depth of 230 nm. A compressive test of the entire tungsten structure was conducted on a universal testing machine (INSTRON 3366) equipped with a 10 kN load cell at a speed of 10 µm s^−1^.

## Conflict of Interest

The authors declare no conflict of interest.

## Supporting information

Supporting Information

## Data Availability

The data that support the findings of this study are available in the supplementary material of this article.
