# Supplementary material for: Fabrication of High‐Density Microarchitected Tungsten via DLP 3D Printing
Source: Adv Sci (Weinh). 2024 Aug 13;11(39):2405487. doi: 10.1002/advs.202405487 (PMC11497019; doi:10.1002/advs.202405487)
Supplement: Supplementary file 1 — Supporting Information [file ADVS-11-2405487-s001.docx]

Supporting Information

Fabrication of High-Density Microarchitected Tungsten via DLP 3D Printing

Junyu Cai, Songhua Ma, Wenbin Yi* and Jieping Wang*

School of Chemistry and Chemical Engineering, Nanjing University of Science and Technology, Nanjing 210094, China
E-mail: yiwb@njust.edu.cn; jieping.wang@njust.edu.cn

**Table S1**. Tungsten-containing organic-inorganic photoresins.

|  | H_2_O (vol%) | PEGDA575 (vol%) | HEA (vol%) | *m*_AMT_ : *m*_resin matrix_ |
| --- | --- | --- | --- | --- |
| R_7:1-50%_ | 20 | 70 | 10 | 0.50 |
| R_7:1-75%_ |  |  |  | 0.75 |
| R_7:1-100%_ |  |  |  | 1.00 |
| R_7:1-125%_ |  |  |  | 1.25 |
| R_7:1-150%_ |  |  |  | 1.50 |
| R_5:3-50%_ | 20 | 50 | 30 | 0.50 |
| R_5:3-75%_ |  |  |  | 0.75 |
| R_5:3-100%_ |  |  |  | 1.00 |
| R_5:3-125%_ |  |  |  | 1.25 |
| R_5:3-150%_ |  |  |  | 1.50 |


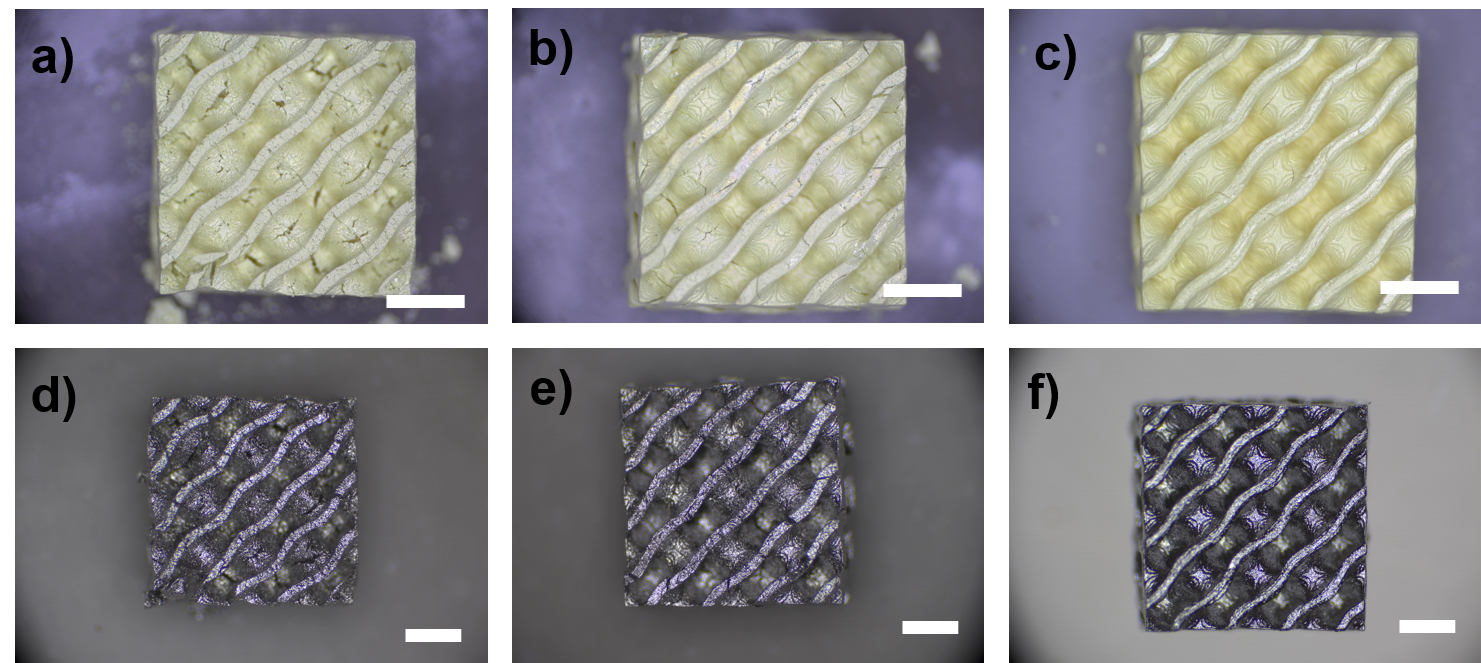


**Figure S1**. a, b, c) Optical images of precursors with varying AMT content (*m*_AMT_ : *m*_resin matrix_ = 0.5:1, 0.75:1, and 1:1, respectively) after debinding in air. d, e, f) Optical images of diamond precursors with varying AMT content (*m*_AMT_ : *m*_resin matrix_=0.5:1, 0.75:1, and 1:1, respectively) after sintering in 10% H_2_-Ar. Scale bars: a, b, c, 2 mm; d, e, f, 1mm.

The optical images revealed that the sample with an AMT and resin matrix mass ratio of 0.5:1 exhibited poor structural integrity after debinding and sintering. However, as the AMT content increased, the samples exhibited superior structural integrity and fewer defects, indicating improved densification.


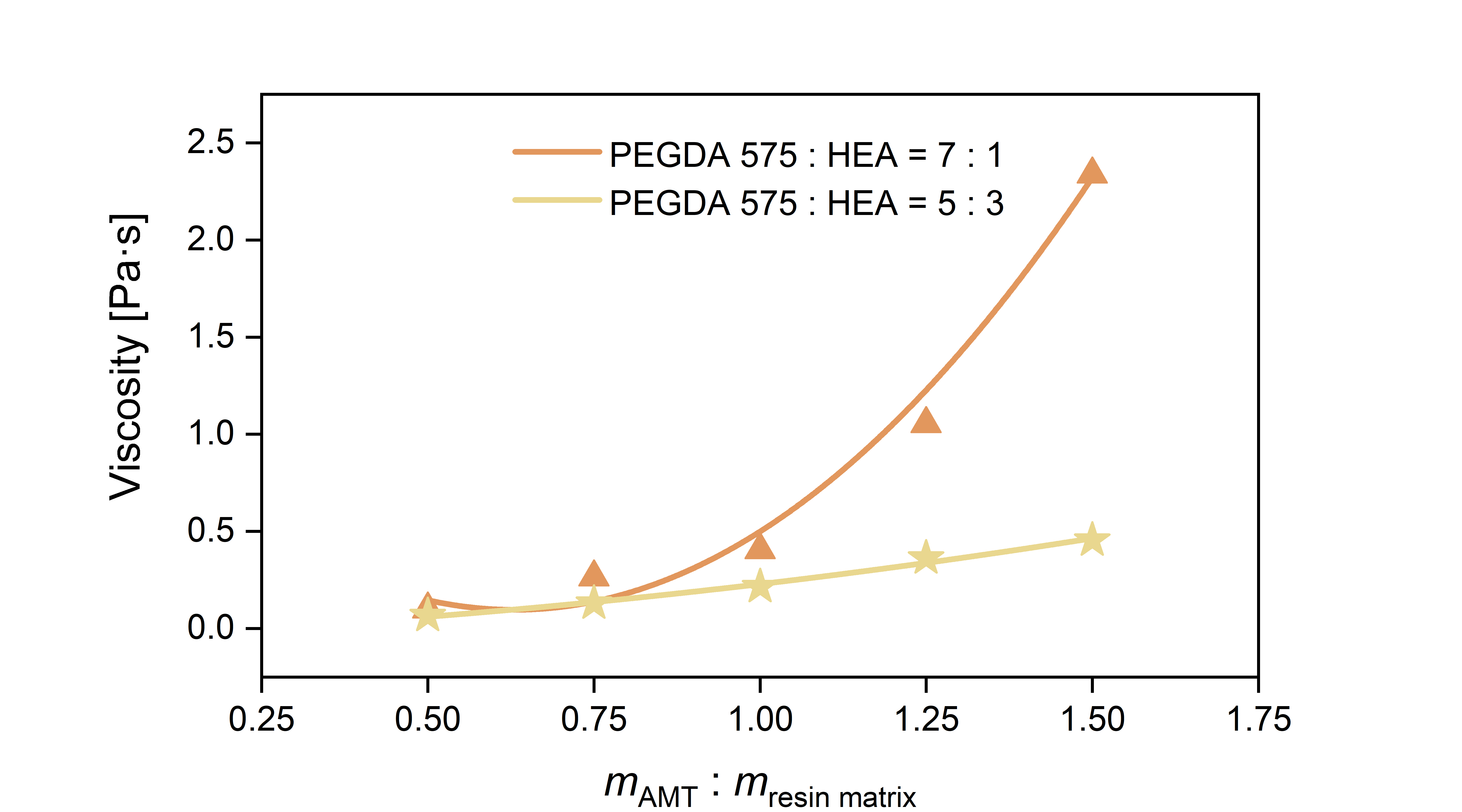


**Figure S2**. The correlation between viscosity and AMT content for photoresins with varying volume ratios of PEGDA 575 and HEA.

With the addition of AMT, the viscosity of both photoresins increased. However, the photoresin with a PEGDA575 to HEA volume ratio of 5:3 exhibited lower viscosity compared to that with a PEGDA575 to HEA volume ratio of 7:1 at the same AMT concentrations. For instance, when the addition of AMT was 1.5 times that of the photoresin matrix, the photoresin with a PEGDA575 to HEA volume ratio of 5:3 exhibited a viscosity of 0.4546 Pa·s, significantly lower than the one with a PEGDA575 to HEA volume ratio of 7:1, which exhibited a viscosity of 2.228 Pa·s. Additionally, when the addition of AMT was 2 times that of the photoresin matrix, the photoresin appeared cloudy, indicating poor compatibility. Consequently, the final composition concentrations of the photoresin matrix were established as 50 vol% PEGDA575, 30 vol% HEA, and 20 vol% D-RO water, and the photoresin was prepared by mixing 60 wt% AMT and 40 wt% photoresin matrix.


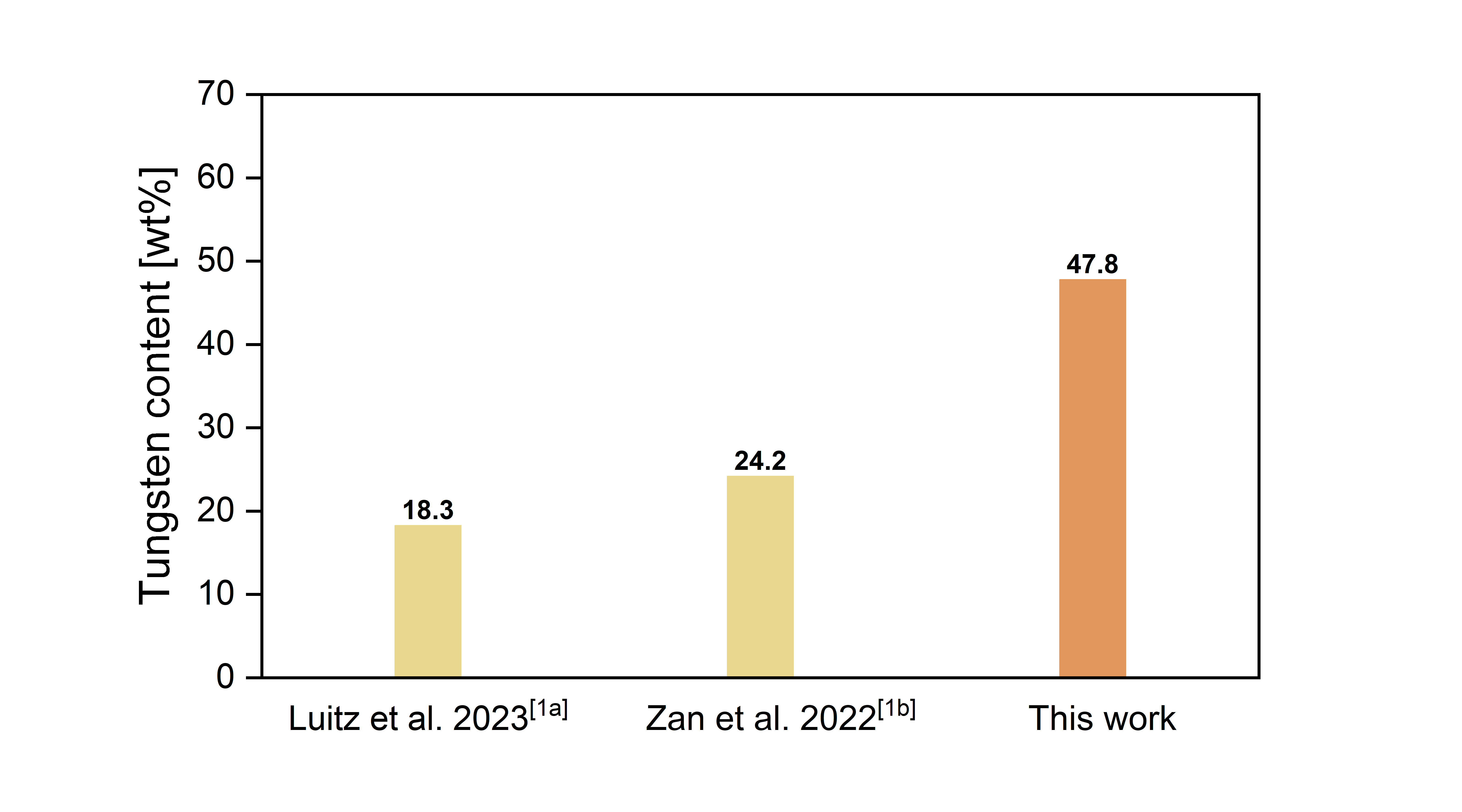


**Figure S3**. Comparison of tungsten loading for related technologies^[1]^.


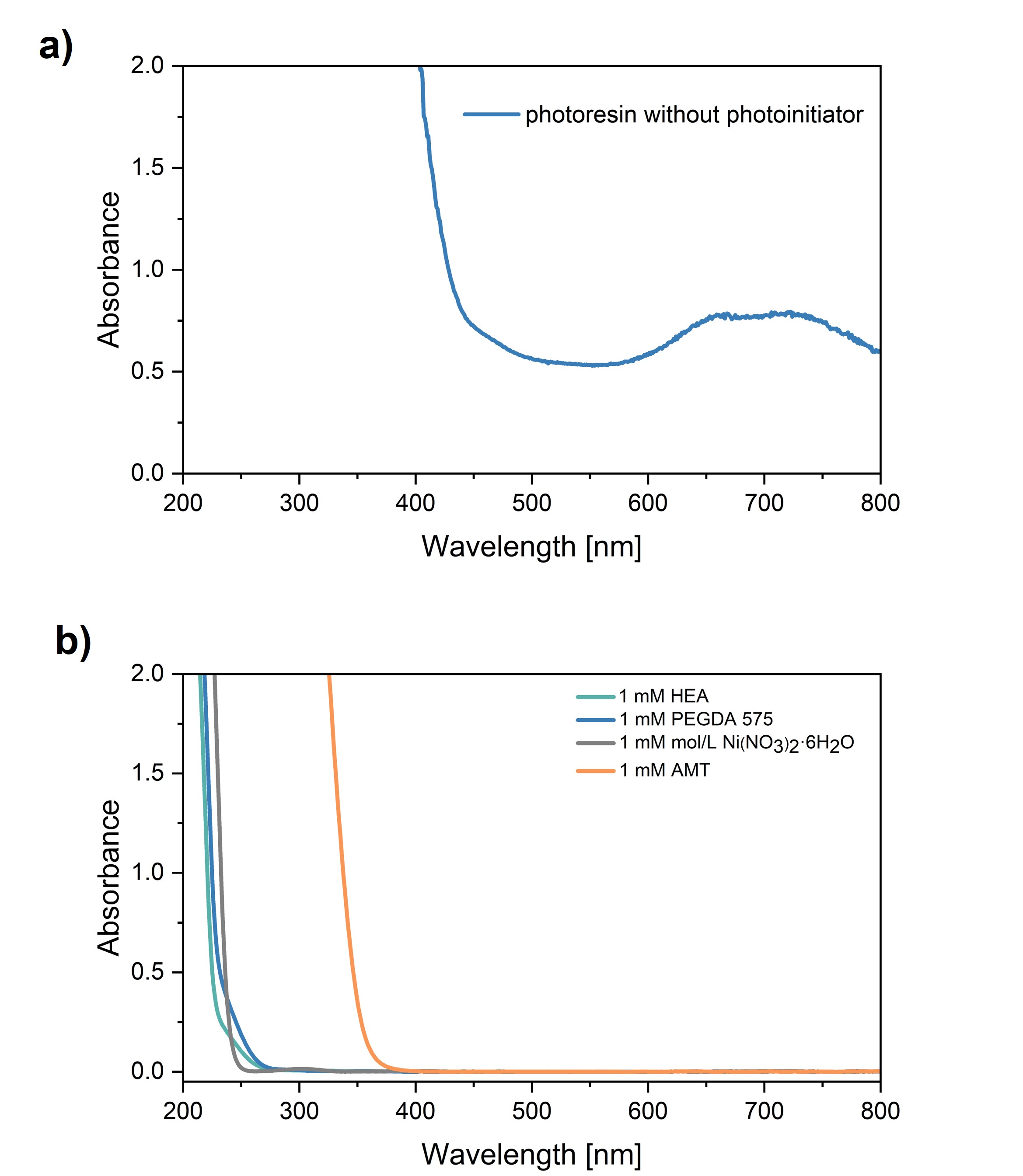


**Figure S4**. UV-Vis absorption spectra of a) the photoresin without photoinitiator and b) the individual photoresin components.


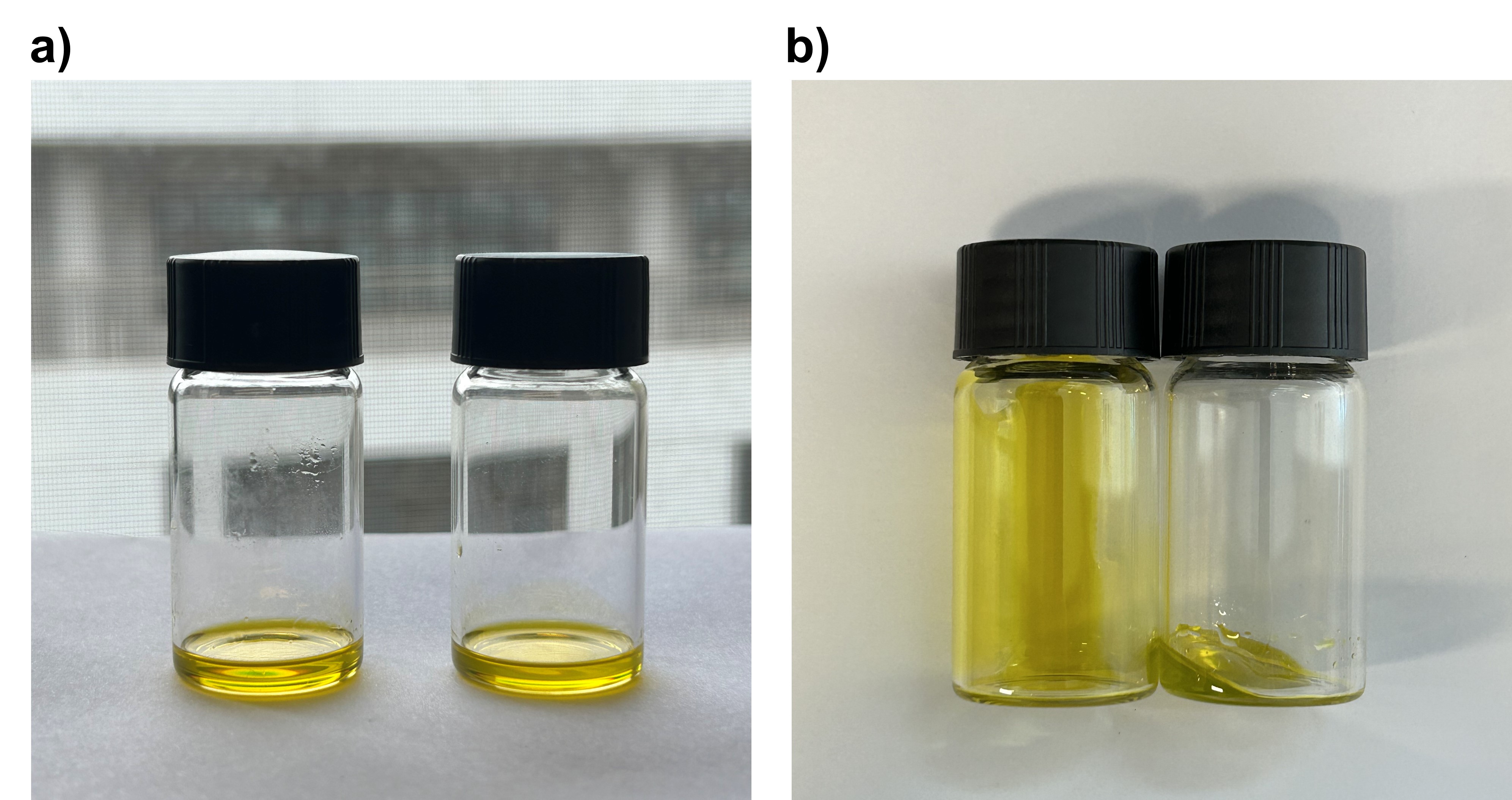


**Figure S5**. Comparative images of photoresins (left: without photoinitiator, right: with photoinitiator) before (a) and after (b) 8-minute UV-LED exposure (385 nm) at 55 mW cm⁻² intensity.


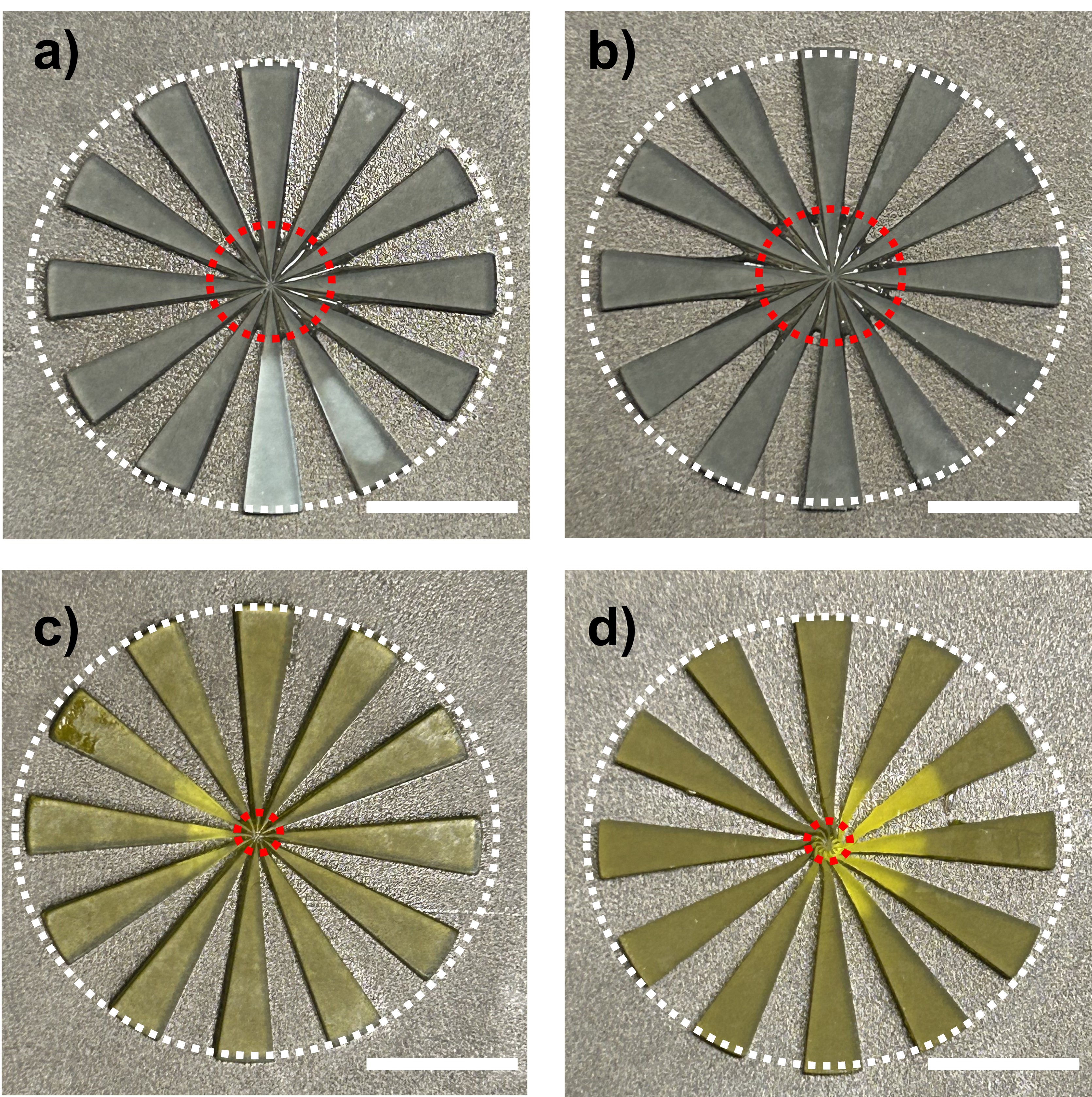


**Figure S6**. Spoke-like patterns (1.2 mm thickness) prepared by photoresins with (c, d) and without (a, b) tartrazine at exposure times of 5 s (a, c) and 10 s (b, d). 10 µm layer thickness, 10 mW cm⁻² intensity. Scale bars: 1 cm.

A spoke-like pattern was employed to assess printing resolution, characterized by the increasing gap between adjacent spokes from the center to the periphery^[2]^. Resolution was quantified as the ratio of unresolved diameter (red dashed circle) to outer diameter (white dashed circle). While the photoresin without tartrazine exhibited a significant resolution variation (20-26%) across different exposure doses, the tartrazine-containing formulation maintained a consistent resolution of 6.1%, independent of exposure conditions.

**Table S2.** Digital light processing parameters.

| Parameter | | Setting |
| --- | --- | --- |
| UV wavelength | | 385 nm |
| Layer thickness | | 10 μm |
| Light intensity | Burn-in | 10 mW cm^-2^ |
|  | Each layer | 10 mW cm^-2^ |
| Exposure time | Burn-in | 6 s |
|  | Each layer | 5.5 s |
| Separation velocity | | 3 mm s^-1^ |
| XY-compensation | | -0.1 – 0.1 mm |
| Wait time (after separation) | | 5 s |


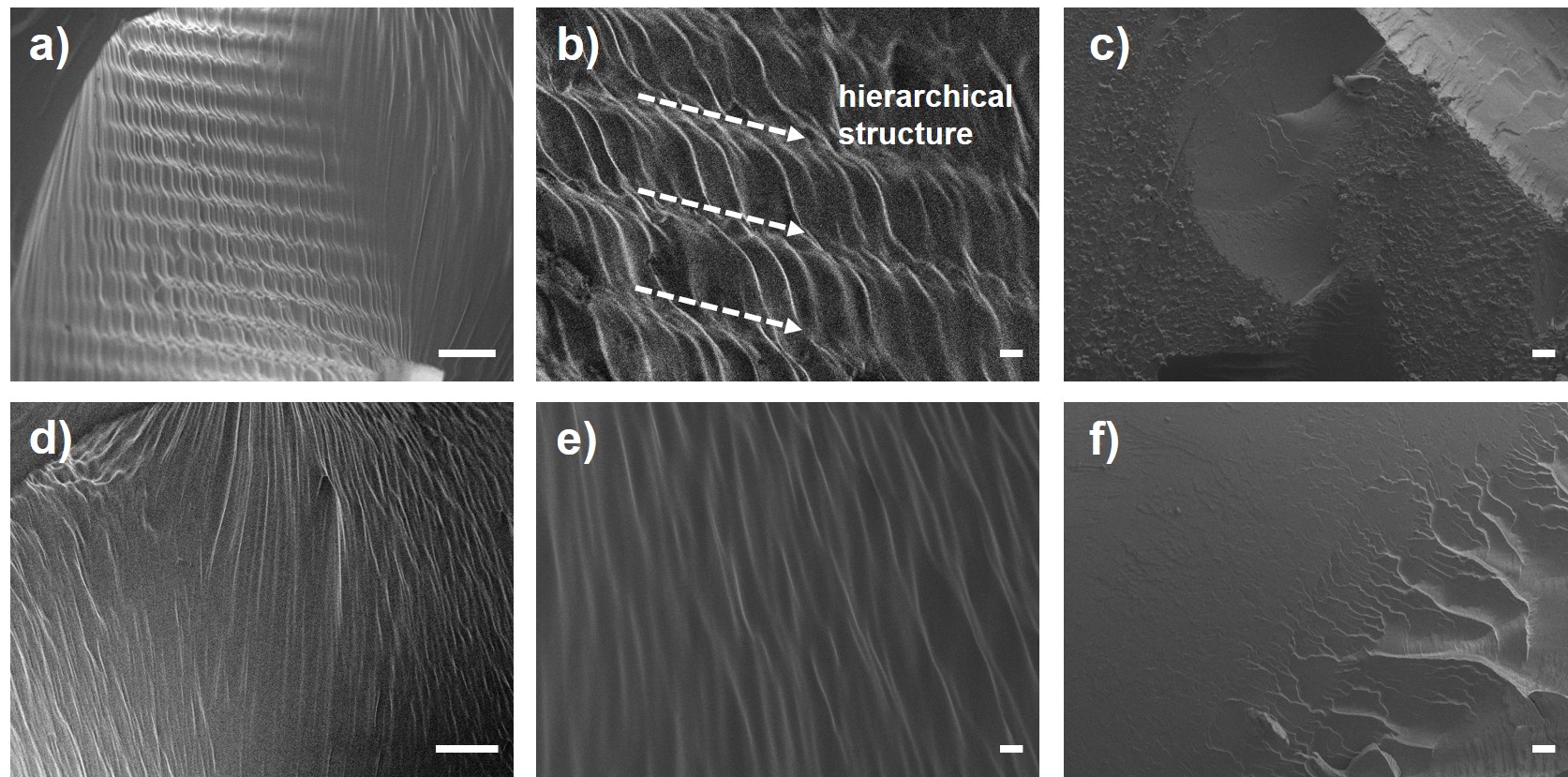


**Figure S7**. SEM images of as-printed precursors with layer thickness of 50 μm (a, b, c) and 10 μm (d, e, f), respectively. a, b, d, e) Surface views of printed precursors. c, f) Cross-section views of printed precursors. Scale bars: a, d, 100 μm; b, e, 10 μm; c, f, 50 μm.


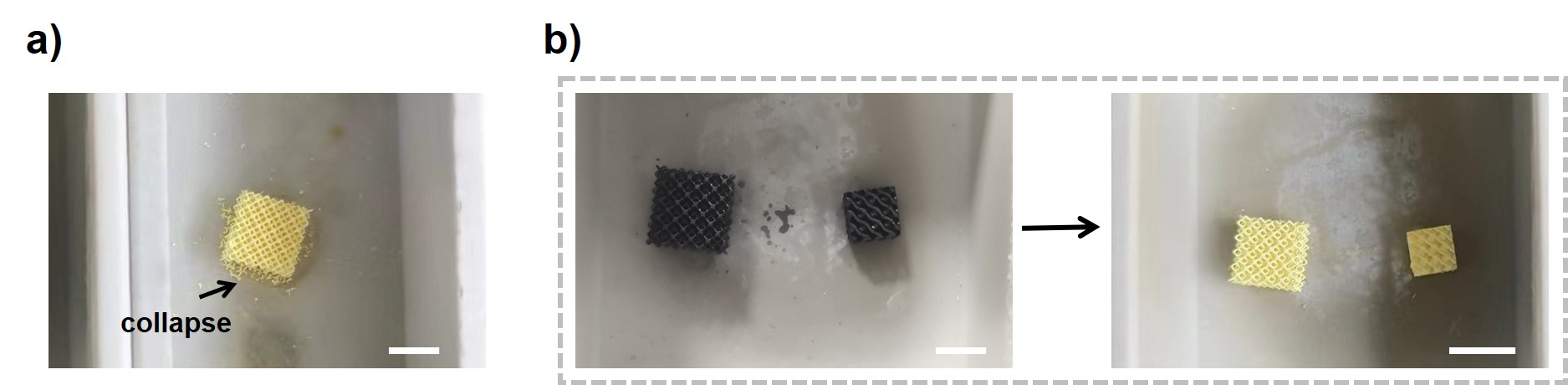


**Figure S8**. Images of as-debinded samples via different debinding strategies. a) Directly debinded under air. b) Pre-debinded under argon (left) followed by completely debinding under air (right). Scale bars: a, b, 5 mm.

Previous research has demonstrated that AMT exhibits similar thermal decomposition behavior in both air and inert atmospheres, reaching complete decomposition at approximately 500 ℃^[3]^. Therefore, we deduced that the difference in weight loss observed between 520 and 550 ℃ was caused by the thermal decomposition of polymers. The presence of oxygen in the air likely intensified the decomposition process.


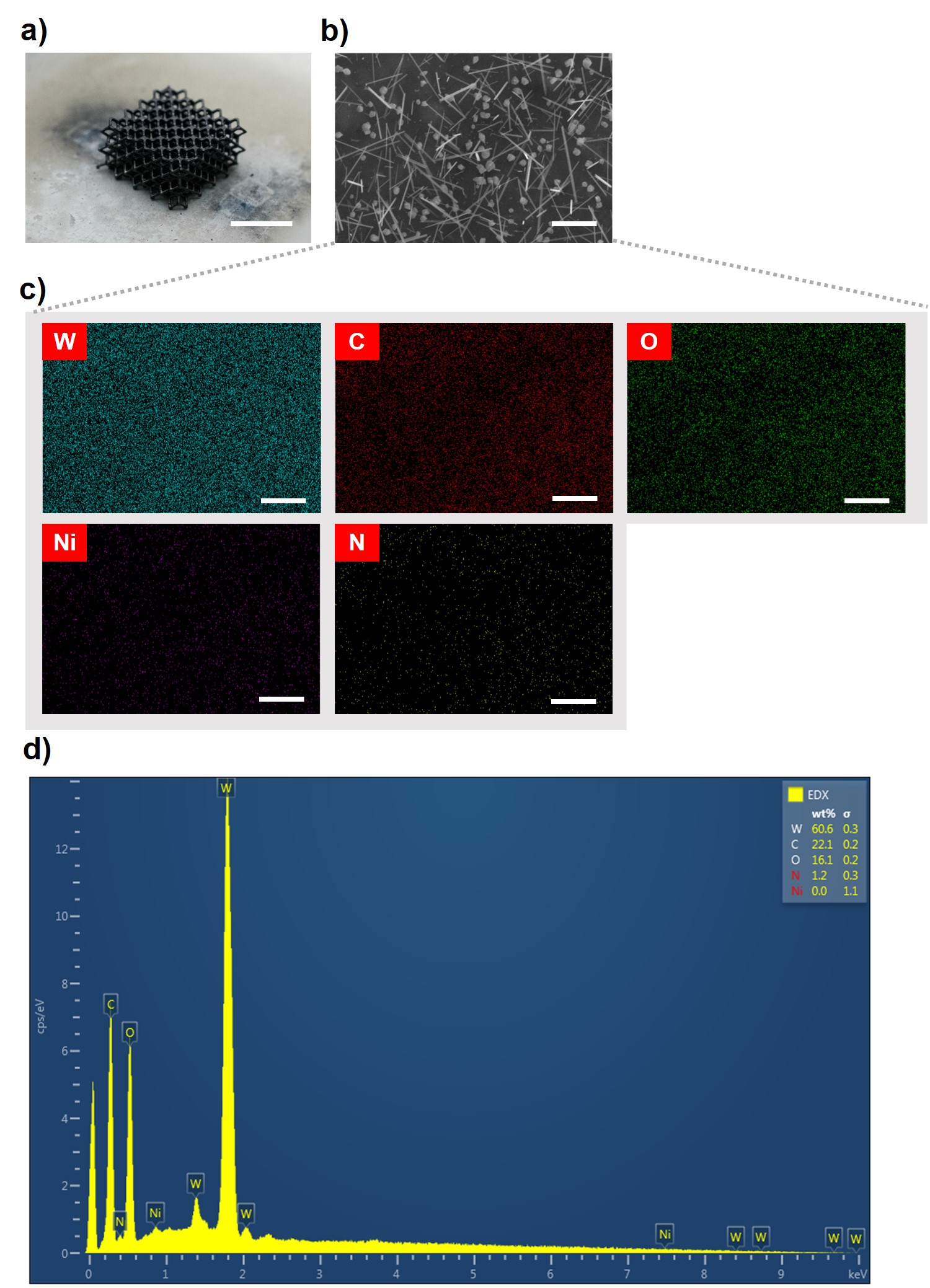


**Figure S9**. a). Optical image of the precursor after debinding in Ar. b) SEM image of the debinded sample. Needle-shaped structures, tentatively identified as WO_3_^[4]^, were observed on the sample surface. c) EDX mapping of the debinded sample. d) EDX spectrum. Scale bars: a, 1 mm; b, c, 2.5 μm.


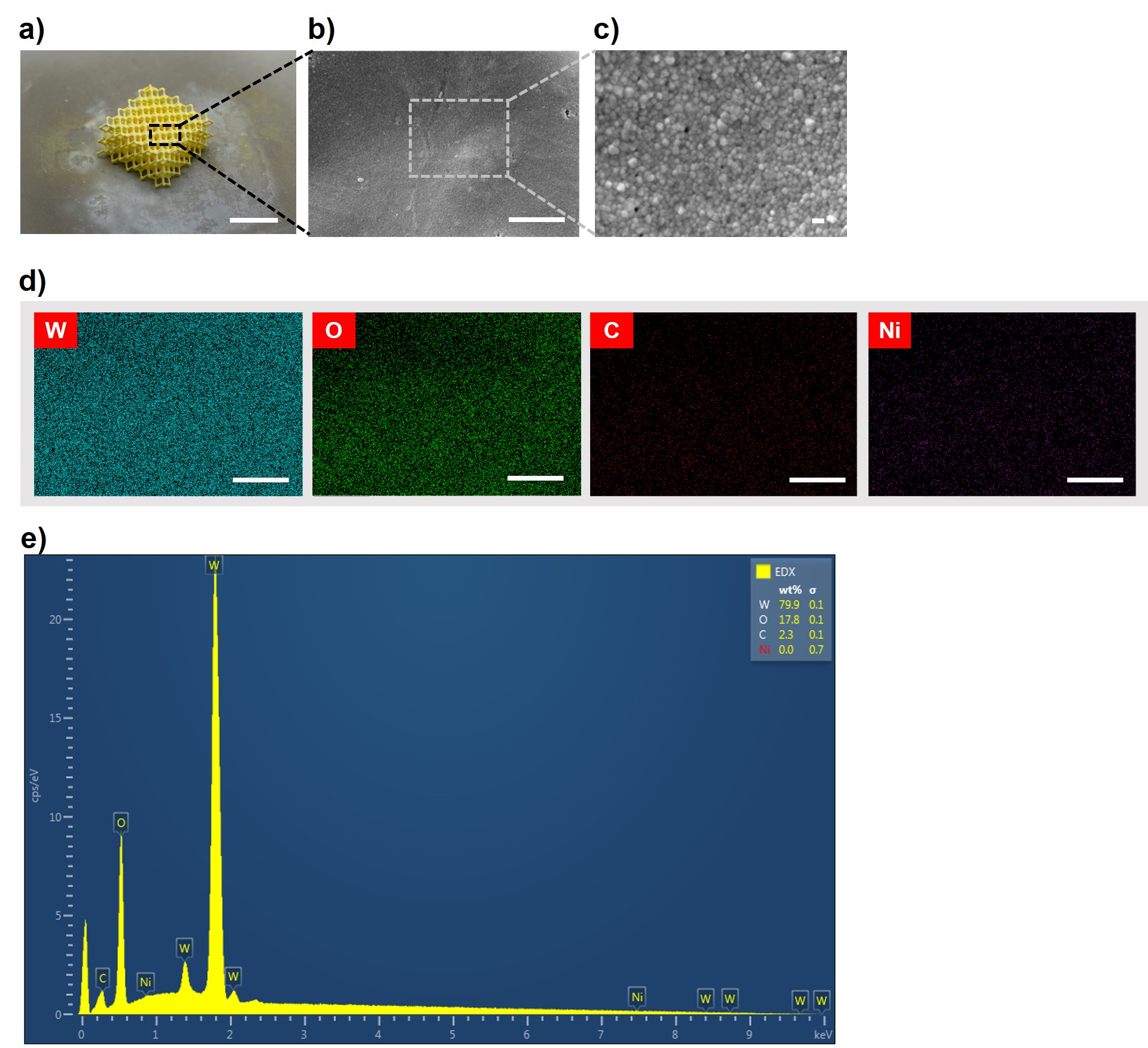


**Figure S10**. a). Optical image of the precursor after debinding in air. b, c) SEM images of the debinded sample. d) EDX mapping of the debinded sample. e) EDX spectrum. Scale bars: a, 5 mm; b, 2.5 μm; c, 100 nm; d, 2.5 μm.


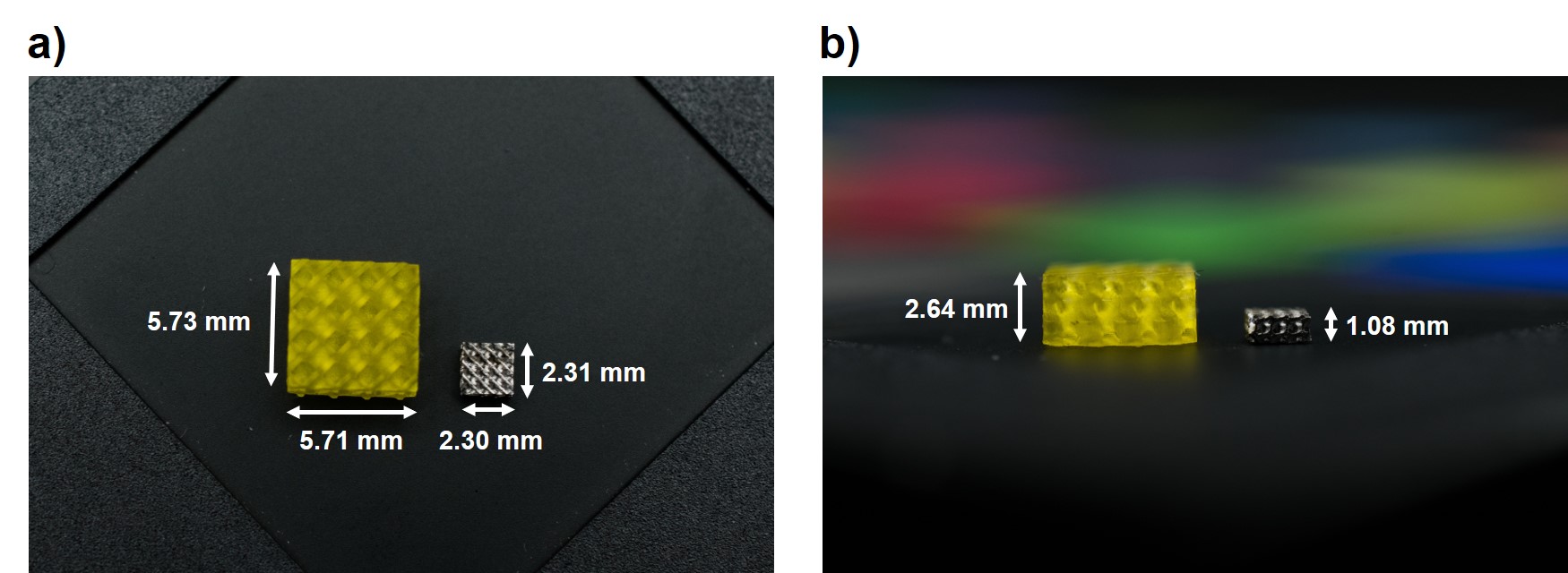


**Figure S11.** The dimensional shrinkage of the fabricated tungsten. a) Dimensional shrinkage in the x and y axes. b) Dimensional shrinkage in the z axes.

**Discussion S1**. Calculation of theoretical linear shrinkage.

The linear shrinkage factor is defined as the ratio of the feature size of the as-sintered tungsten to that of the tungsten-contained precursor, serving as an indicator for densification. A lower linear shrinkage factor indicates a decreased presence of residual pores following debinding and sintering. The theoretical linear shrinkage factor can be calculated by making the following assumptions: 1) AMT is fully converted to tungsten. 2) The polymers are completely decomposed. 3) The shrinkage is isotropic. Base on the above assumptions, the linear shrinkage factor can be approximated as:

$$Linear shrinkage factor=\sqrt[3]{\frac{V_{W}}{V_{P}}} \times100\%$$

where *V*_W_ is the volume of the fabricated tungsten and *V*_P_ is the volume of the tungsten-containing precursor. *V*_W_ can be calculated as:

$$V_{W}=\frac{m_{W}}{\rho_{W}}$$

where *m*_W_ is the mass of tungsten in the tungsten-containing precursor and *ρ*_W_ is the density of tungsten. *m*_W_ can be calculated as:

$${m_{W}=m}_{P}\times x_{W}$$

where *m*_p_ is the mass of tungsten-containing precursor and *x*_W_ is the mass fraction of tungsten in printed precursor. Thus, the linear shrinkage factor can be approximated as:

$$Linear shrinkage factor=\sqrt[3]{\frac{m_{P} \times x_{W}}{V_{P} \times\rho_{W}}}\times100\%$$

In this work, *ρ*_W_ is taken as 19.35 g cm^-2^ and *x*_W_ is 47.8%, corresponding to the tungsten content in the photoresin. A block precursor with a volume of 93.5 mm^3^ (7.57 mm × 7.62 mm × 1.62 mm) and a mass of 0.2174 g was printed. According to the equation derived above, the theoretical linear shrinkage factor was determined to be 38.5%.


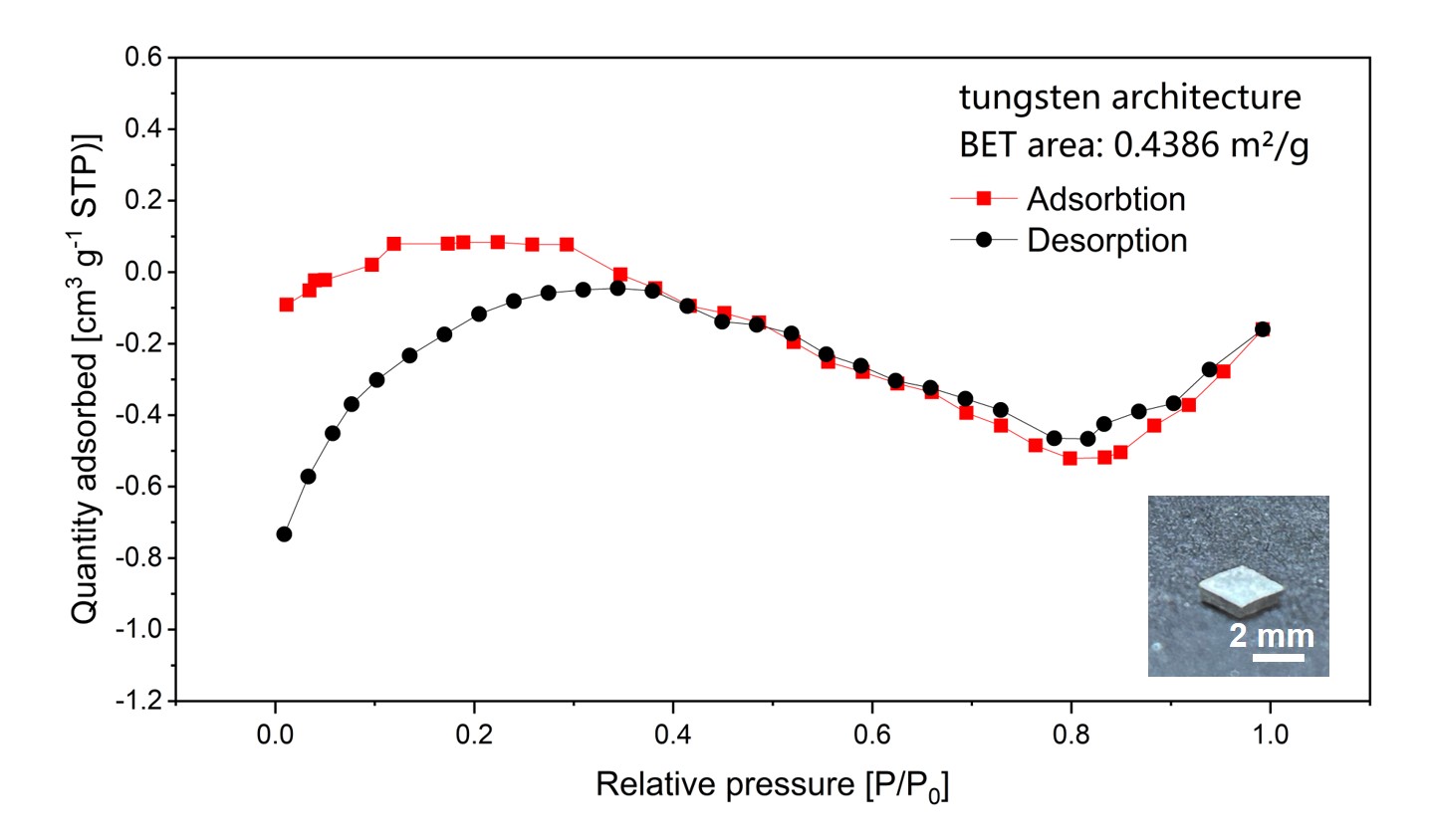


**Figure S12**. Brunauer-Emmett-Teller (BET) analysis of the fabricated tungsten.

**
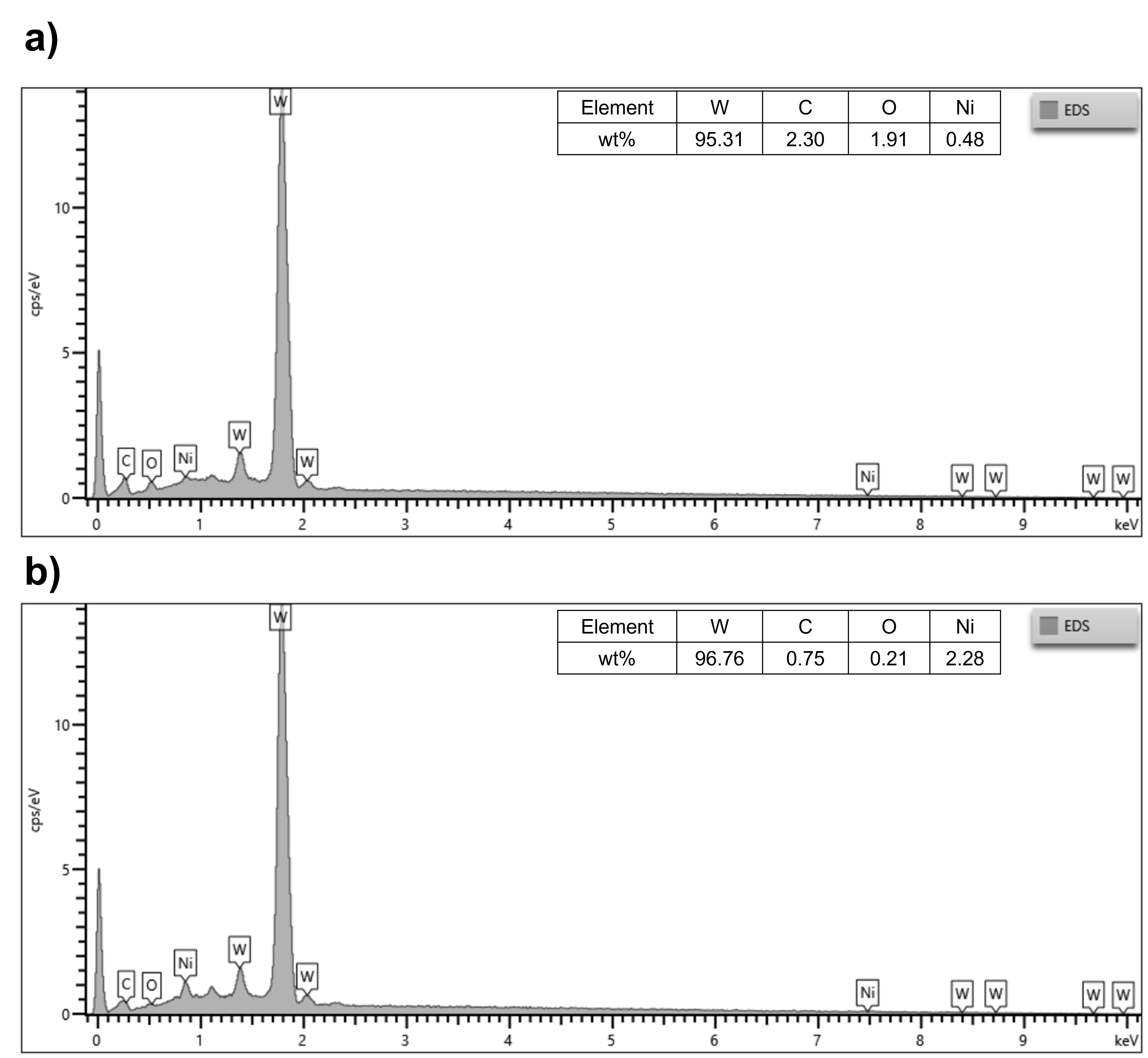
**

**Figure S13**. a) EDX spectrum of the fabricated tungsten surface. b) EDX spectrum of the fabricated tungsten cross-section.

**
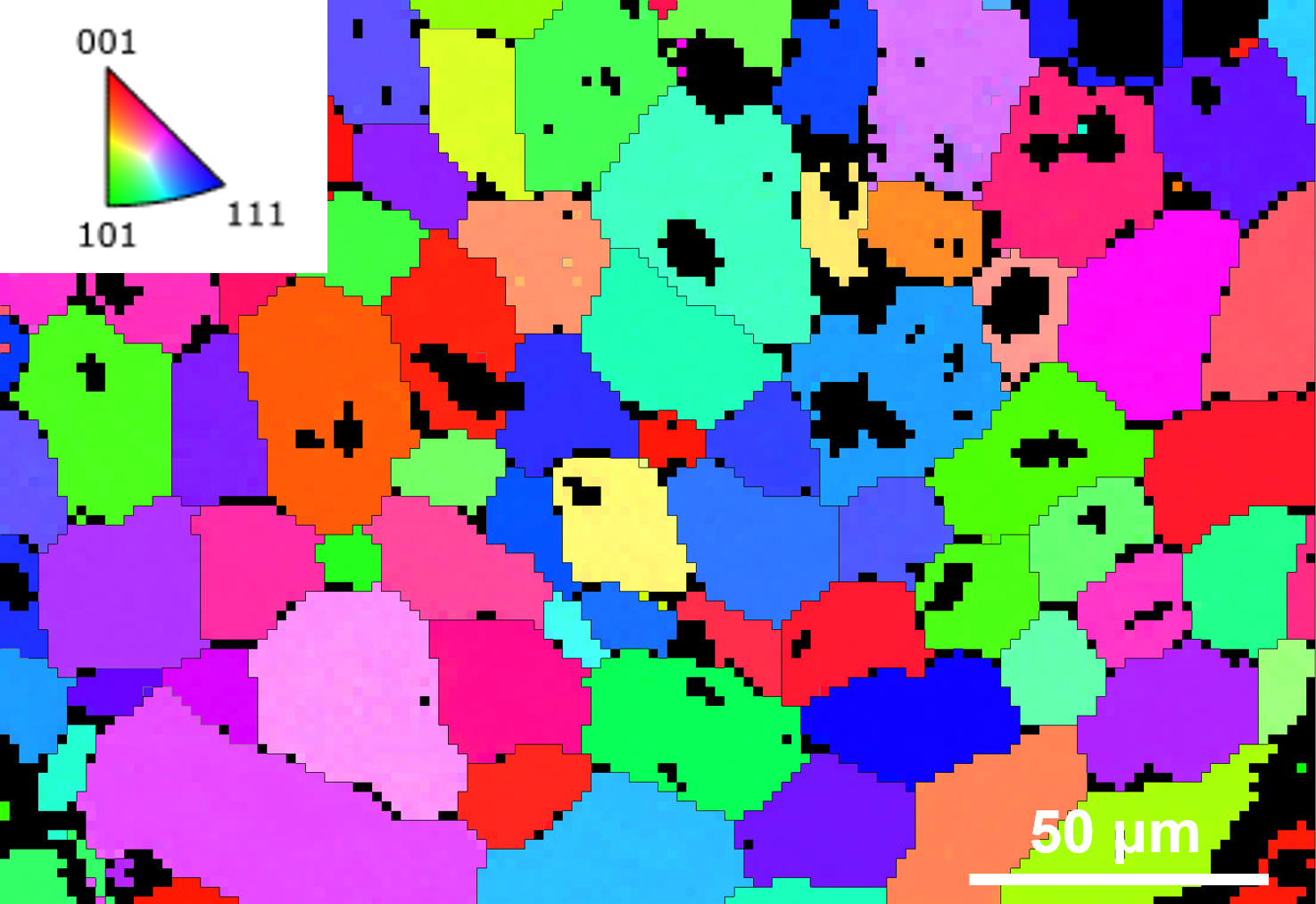
**

**Figure S 14.** EBSD image of the fabricated tungsten.

**Table S3.** Grain size statistics obtained from EBSD.

| Avg. grain size [µm] | Area-weighted Avg. grain [µm] | Standard deviation [µm] | Count |
| --- | --- | --- | --- |
| 18.8 | 24.8 | 7.9 | 79 |


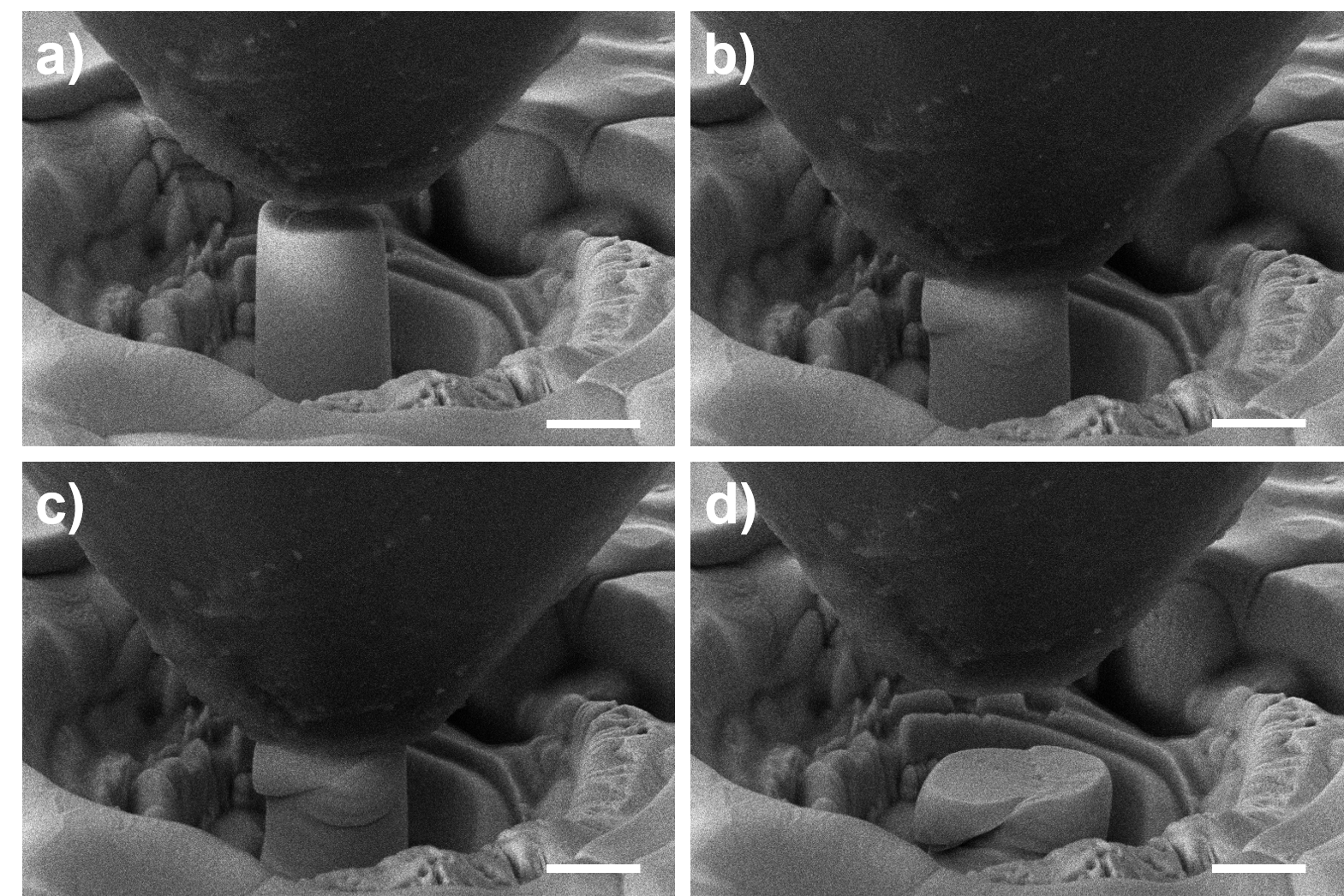


**Figure S15**. Images of micropillar compression captured from a real-time monitoring video during the micropillar compression process. a) The beginning of compression. b) The micropillar begins to deform. c) Severe deformation of the micropillar. d) The end of compression. Scale bar: a, b, c, d, 5 μm.


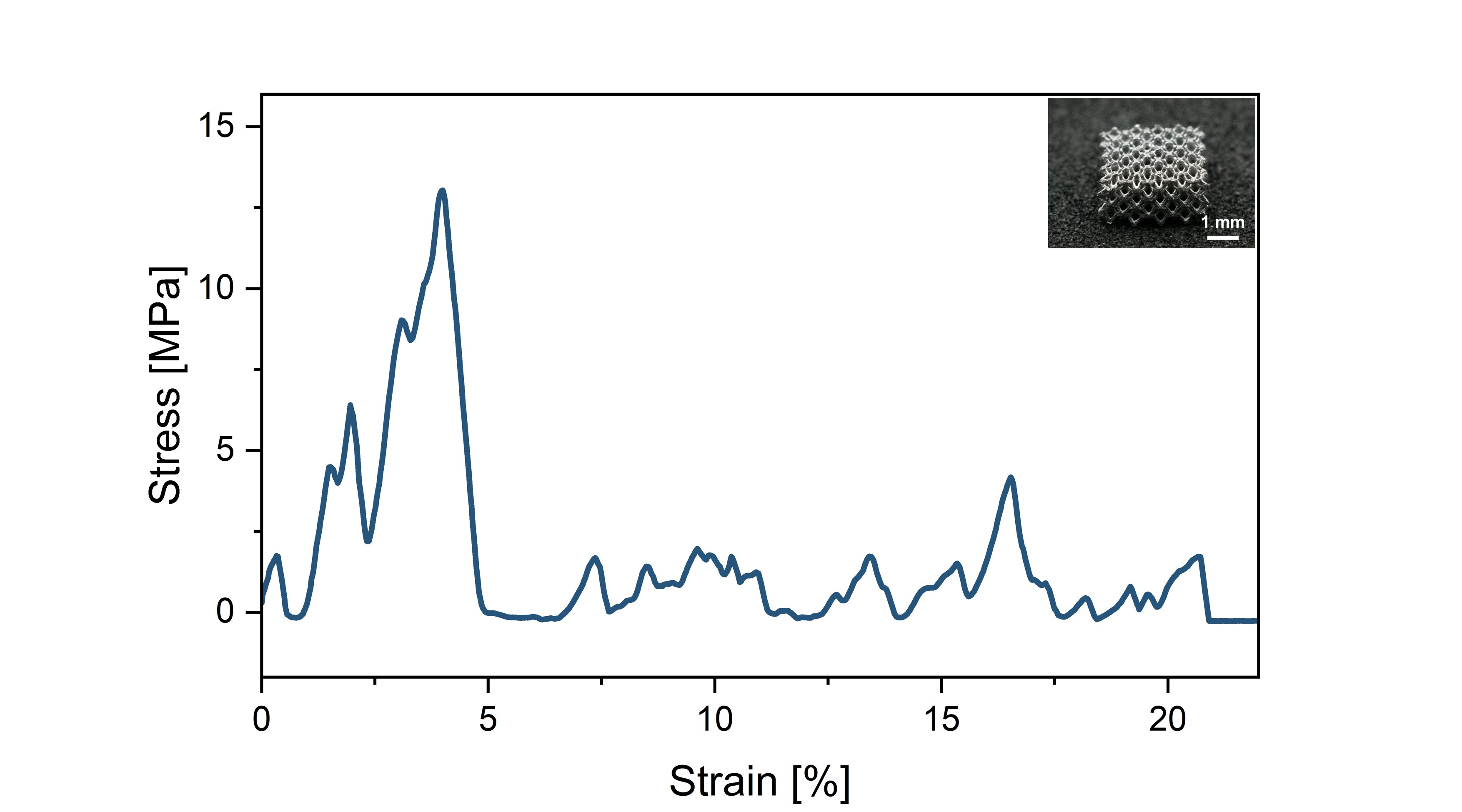


**Figure S16**. Compressive stress-strain curve of the fabricated tungsten lattice.

**Table S4**. Reference data for tungsten micropillar compression.

| σ at 2.5% strain (MPa) | D [nm] | ρ | Reference |
| --- | --- | --- | --- |
| 2800 | 200 | 0.16 | [5] |
| 1650 | 4000 |  |  |
| C=6378 | | | |

**Discussion S2**. The predicted yield strength for a tungsten micropillar.

The strength of face-centered cubic (fcc) and body-centered cubic (bcc) micropillars obeys the power-law relationship ^[6]^:

$$\sigma=C \times D^{-\rho}$$

where *σ* is the yield strength or flow stress, *C* is a constant, *D* is the micropillar diameter, and *ρ* is the power-law exponent. Schneider et. al. revealed a fit line of $\sigma\propto D^{-0.16}$ for tungsten micropillars^[5]^. Based on the data in Table S3, the constant *C* was established as 6378. The predicted yield strength for a tungsten micropillar with a diameter of 6 μm was 1585 MPa, which closely matched the experimental value of 1552 MPa.

**Table S5.** Nanoindentation hardness and Young's modulus (E_r_) of the fabricated tungsten measured by load-controlled mode.

|  | Contact depth [nm] | Hardness [GPa] | E_r_ [GPa] |
| --- | --- | --- | --- |
| 1 | 185.2 | 8.6 | 257.6 |
| 2 | 195.5 | 7.8 | 301.7 |
| 3 | 206.8 | 7.0 | 284. 7 |
| 4 | 195.8 | 7.8 | 316.4 |
| 5 | 206.4 | 7.1 | 297.8 |
| 6 | 211.9 | 6.7 | 308.5 |
| 7 | 209.2 | 6.9 | 303.4 |
| 8 | 198.2 | 7.6 | 272.6 |
| 9 | 189.1 | 8.3 | 287.4 |
| Average | 199.8 | 7.5 | 292.2 |

**Table S6.** Reference data for nanoindentation of tungsten.

| Depth [nm] | Hardness [GPa] | Young's modulus [GPa] | Reference | This work [GPa] | H_W_/H_R_ |
| --- | --- | --- | --- | --- | --- |
| 40 - 100 | 8.63 | 270 | [7] | —— | —— |
| 150 | 4.2 | 309 | [8] | 8.60 | 2.05 |
| 30 | 7.82 | 270.2 | [9] | 11.60 | 1.48 |
| 190 | 6.36 | 259.8 |  | 7.96 | 1.25 |
| 100 - 200 | 6.0 | N/A | [10] | —— | —— |
| 45.9 ± 1.4 | 5.45 ± 0.25 | 322 ± 36 | [11] | 10.60 | 1.94 |
| 36.3 ± 2.2 | 7.84 ± 0.72 | 345 ± 43 |  | 11.32 | 1.44 |
| 31.0 ± 1.2 | 6.91 ± 0.34 | 325 ± 39 3 |  | 11.46 | 1.66 |
| 26.1 ± 2.4 | 8.73 ± 1.1 | 335 ± 47 |  | 11.87 | 1.36 |

**References**

[1] a) X. Zan, X. Wang, K. Shi, Y. Feng, J. Shu, J. Liao, R. Wang, C. Peng, S. Magdassi, X. Wang, *J. Phys. D: Appl. Phys.* **2022**, 55, 444004; b) M. Luitz, D. Pellegrini, M. von Holst, K. Seteiz, L. Gröner, M. Schleyer, M. Daub, A. Warmbold, Y. Thomann, R. Thomann, *Adv. Eng. Mater.* **2023**, 25, 2201927.

[2] S. You, P. Wang, J. Schimelman, H. H. Hwang, S. Chen, *Addit. Manuf.* **2019**, 30, 100834.

[3] D. Hunyadi, I. Sajó, I. M. Szilágyi, *J. Therm. Anal. Calorim.* **2014**, 116, 329.

[4] a) Q. Hu, J. He, J. Chang, J. Gao, J. Huang, L. Feng, *ACS Appl. Nano Mater.* **2020**, 3, 9046; b) M. U. Qadri, T. Stoycheva, M. C. Pujol, E. Llobet, X. Correig, J. F. Borull, M. Aguiló, F. Díaz, *Procedia Eng.* **2011**, 25, 761.

[5] A. Schneider, C. Frick, B. Clark, P. Gruber, E. Arzt, *Mater. Sci. Eng., A* **2011**, 528, 1540.

[6] S.-W. Lee, W. D. Nix, *Philos. Mag.* **2012**, 92, 1238.

[7] H. Huang, Y. Wu, S. Wang, Y. He, J. Zou, B. Huang, C. Liu, *Mater. Sci. Eng., A* **2009**, 523, 193.

[8] W. W. Gerberich, W. Yu, D. Kramer, A. Strojny, D. Bahr, E. Lilleodden, J. Nelson, *J. Mater. Res.* **1998**, 13, 421.

[9] Z. Wen, G. Xuanqiao, Z. Pingxiang, H. Zhongwu, L. Laiping, C. Jun, *Rare Met. Mater. Eng.* **2017**, 46, 3626.

[10] D. Armstrong, P. Edmondson, S. Roberts, *Appl. Phys. Lett.* **2013**, 102, 251901.

[11] B. D. Beake, S. Goel, *Int. J. Refract. Met. Hard Mater.* **2018**, 75, 63.
